# A Cost-Effectiveness and Quality of Life Analysis of Different Approaches to the Management and Treatment of Localized Prostate Cancer

**DOI:** 10.3389/fonc.2020.00103

**Published:** 2020-02-11

**Authors:** Aleksandra Harat, Maciej Harat, Melissa Martinson

**Affiliations:** ^1^Department of Social and Medical Sciences, Ludwik Rydygier Collegium Medicum, Nicolaus Copernicus University, Bydgoszcz, Poland; ^2^Department of Oncology and Brachytherapy, Ludwik Rydygier Collegium Medicum, Nicolaus Copernicus University, Bydgoszcz, Poland; ^3^Department of Radiotherapy, Franciszek Lukaszczyk Oncology Center, Bydgoszcz, Poland; ^4^Technomics Research, Minneapolis, MN, United States

**Keywords:** active monitoring, cost-effectiveness analysis, prostate cancer, prostatectomy, QALY, radiotherapy

## Abstract

The aim of this study was to compare the cost-effectiveness and quality-adjusted life years (QALYs) of active monitoring (AM), radical prostatectomy (PR), and external-beam radiotherapy with neoadjuvant hormone therapy (RT) for localized prostate cancer. Microsimulations of radical prostatectomy, 3D-conformal radiotherapy, or active monitoring were performed using Medicare reimbursement schedules and clinical trial results for a target population of men aged 50–69 years with newly diagnosed localized prostate cancer (T1-T2, NX, M0) over a time horizon of 10 years. Quality-adjusted life years (QALYs) and costs were assessed and sensitivity analyses performed. Monte Carlo simulations revealed that the mean cost for AM, PR, and RT were $15,654, $18,791, and $30,378, respectively, and QALYs were 6.96, 7.44, and 7.9 years, respectively. The incremental cost-effectiveness ratio (ICER) was $6,548 for PR over AM and $68,339 for RT over PR. Results were sensitive to the number of years of follow-up and procedure cost. With relaxed assumptions for AM, the ICER of PR and RT met the societal willingness to pay (WTP) threshold of $50,000 per QALY. Compared with AM, PR was highly cost-effective. RT and PR for localized prostate cancer can be cost-effective, but RT must offer increased QALYs or decreased procedural costs to be cost-effective compared to PR. Newer and cheaper radiotherapy strategies like stereotactic body radiotherapy may play a crucial role in future early prostate cancer management.

## Introduction

About 160–240,000 men are diagnosed with prostate cancer in the US each year (1, 2). Prostate cancer has a tremendous and growing economic impact in part due to the costs associated with newer therapies. There is, however, no consensus on the most cost-effective treatment strategy for low- and favorable-risk prostate cancer.

The Prostate Testing for Cancer and Treatment (ProtecT) trial examined the optimal management of men with low-risk, clinically localized prostate cancer detected by prostate serum antigen (PSA) testing by comparing active monitoring (AM), radical prostatectomy (PR), and external-beam radiotherapy with neoadjuvant androgen deprivation therapy (RT). ProtecT reported no significant differences in prostate cancer-specific mortality or all-cause mortality at a median follow-up of 10 years regardless of strategy. Although the trial revealed worse outcomes for AM in terms of disease progression and metastasis, ProtecT clarified the distinct effects of prostate cancer treatments on urinary, sexual, and bowel function and condition-specific quality of life (QoL) (3, 4).

Differences between treatment modalities in terms of side-effects and costs may translate into more or less cost-effective management. The most recent cost-effectiveness analyses comparing AM with immediate treatment (5) or primary treatments for clinically localized prostate cancer (6) were evaluated before ProtecT reported. The estimates were based on a large systematic review of lower-level evidence and were thus limited by the quality and quantity of data (5).

The aim of this cost-effectiveness study was to estimate the long-term health outcomes and healthcare costs of the three localized prostate cancer treatment strategies used in ProtecT. The study leverages the results of this first multicenter randomized trial and accounts for cost and risk of death, recurrence, salvage therapy, adverse effects, and complications related to treatment.

## Methods

### Study Design and Scope

**Figure 1:**
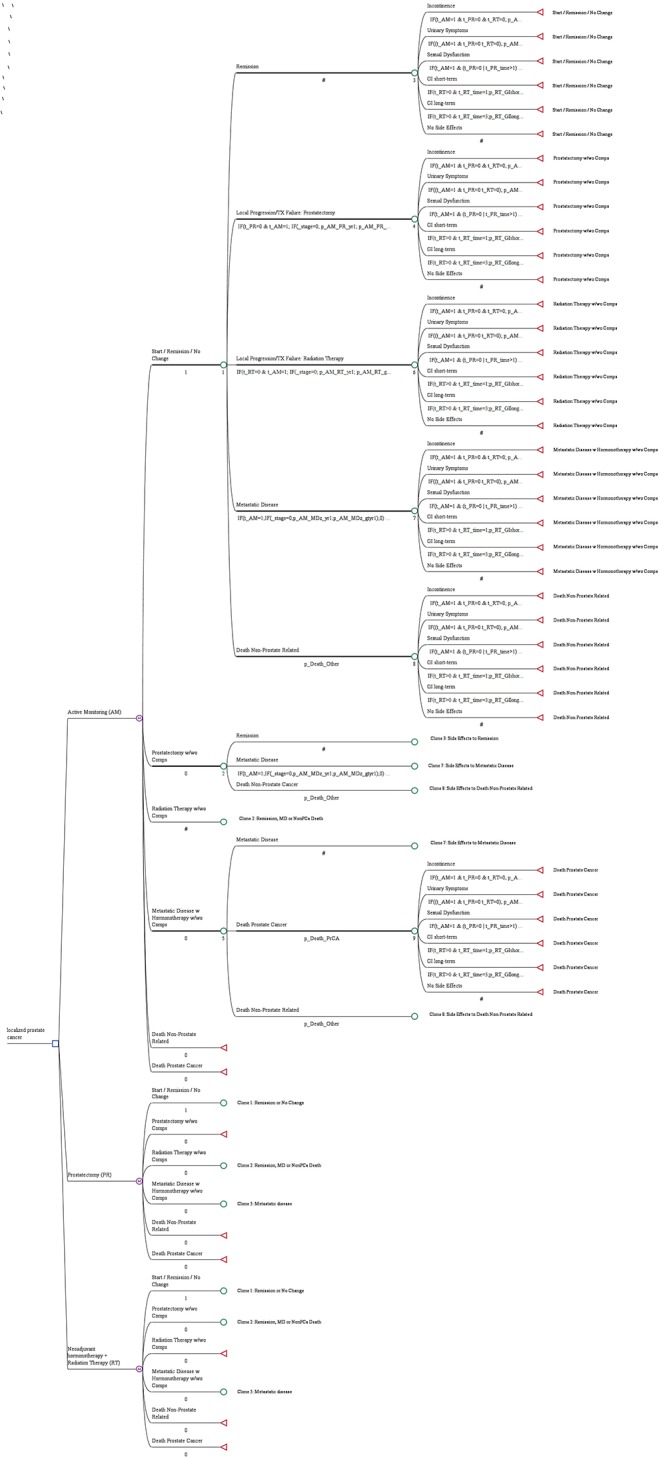
A decision tree for managing newly diagnosed prostate cancer. The blue square indicates a decision node, a point at which a treatment strategy is chosen; the purple encircled letter “M” indicates the Markov node, with branches indicating the health states in transition every 6 months; the green circle indicates the chance node, after which there is a probability of the occurrence of each health state (remission, local progression treated with prostatectomy, local progression treated with radiation therapy, metastatic disease, death not prostate related, death from prostate cancer); and the red triangle indicates the terminal node, the end of a pathway within a 6 months cycle.

A Markov model of managing newly diagnosed prostate cancer was developed using TreeAge Software (TreeAge Software Inc., Williamstown, MA; Figure 1). Monte Carlo simulations were performed to estimate the costs and QALYs of patients with histologically proven, clinically localized prostate cancer (T1-T2, NX, M0) over the 10 years from diagnosis in 6 months increments (stages). Costs of diagnosis were not included because they were treatment-independent. The analysis was conducted from the US healthcare payer perspective, with national-average Medicare reimbursements for year 2008 used as payer costs. In accordance with economic guidelines, the 3% discount rate was used to adjust costs to their net present value.

The analysis included three prostate cancer treatments: active monitoring (AM), prostatectomy (PR), and external beam radiotherapy (RT). Health states for each stage were remission, local progression, metastatic disease, and prostate cancer-related and non-prostate cancer-related deaths. Cost analyses did not include patients that did not start any treatment or started another form of treatment in the ProtecT trial. To exclude protocol-driven costs (7), we verified the protocol according to well-established National Comprehensive Cancer Network (NCCN) recommendations (8).

Simulations of various scenarios to estimate cost of treatment of clinically localized prostate cancer (PSA level <20, Gleason 6–10, stage ≤ T2) were conducted. Men entered the model aged 50–69 and exited at the time of death or after 10 years of follow-up.

The decision tree in Figure 1 shows the microsimulation model used to simulate costs and quality-adjusted life-years (QALYs). According to the study profile, the three groups (AM, PR, and RT) were analyzed, and the decision tree considered three health states (progression-free survival, progressive disease, and death). The target population was a hypothetical cohort of 545 people with the same characteristics as those in ProtecT.

### Model Inputs

Treatment scenarios, group sizes, and cost centers were generated based on the original study results (3, 4). Other phase three randomized trials on active surveillance for localized prostate cancer (3, 9–12) were used to predict missing cost centers, incidence of events, and treatment results not reported in Hamdy et al. (3) and validated by expert panels.

A previous decision analysis of the ProtecT trial was used to estimate QALYs (13). Model inputs are described in detail in Supplementary Table 1. To standardize costs, we derived unit and resource costs from the Medicare Fee Schedule for the Technical Component of Hospital Outpatient Radiology Procedures (14, 15).

Sensitivity analyses were undertaken to handle parameter uncertainty. Specific analytic assumptions about the variation in costs and outcomes were made in order to obtain confidence intervals on cost effectiveness ratios (16). Sensitive parameters taken into account were number of follow-up years, specific costs, and probabilities.

For the purpose of this study, we assumed that patients underwent prostatectomy via the conventional retropubic approach. The risk of post- or peri-operative complications per model stage was set at 7.5% for urinary symptoms, 22% for incontinence, and 27% for sexual dysfunction for all patients undergoing prostatectomy (3). In our scenario, the frequency of minor vs. major surgical complications was assumed to be 2:1 based on Institute for Clinical and Economic Review (14, 15). The corresponding probabilities in the AM treatment were 2.5, 0.5, and 2.6% and in the RT treatment were 4.6, 0.3, and 20.5%. In addition, patients receiving RT risked short- (2.5%) and long-term (3.6%) gastrointestinal problems.

For each treatment, specific costs and management of treatment-related adverse effects were derived from Institute for Clinical and Economic Review (14, 15) and Hodges et al. (17), and the numbers of patients with treatment-related adverse effects were extracted from patient-reported outcomes (4) and long-term functional outcome data (18). The number of patients that received treatment-related negative effects was calculated based on the following formula: max % of patients that reported negative effect—% of patients with negative effect at baseline x number of patients treated.

We calculated the ICERs expressed as monetary costs per life-years gained (LYG) and per QALYs gained, and compared each to the cost-effectiveness threshold, which represents society's willingness to pay (WTP) for an additional unit of benefit. In the US, the commonly accepted standard threshold is $50,000 per QALY gained.

One-way sensitivity analysis was performed for all parameters to assess the impact that a fixed change in each parameter had on the ICER. A cost-effectiveness acceptability curve was constructed to determine the probability of each strategy of being cost-effective. The multivariate probabilistic analysis was performed running 1,000 patients in 10,000 Monte Carlo iterations. Since this was a secondary analysis of anonymized data, no IRB approval was required.

## Results

### Model Validation

The difference in survival benefit in ProtecT was not significant between the AM, PR, and RT groups, but the distant metastasis and progression rates were higher in the AM group. The model accurately reproduced the survival outcomes of ProtecT in terms of overall undiscounted survival over a 10 years period: PR average 9.57 life years, RT average 9.57 life years when rounded, but slightly <PR, and AM average 9.53 life years.

### Cost and Life Years as an Effectiveness Measure

After applying a 3% annual discount rate, RT was the most expensive at $30,378 over 10 years. Since RT was equally effective as PR but also more expensive than PR at $18,791, PR could be regarded the better choice. Both PR and AM represent rational choices, because AM is less effective and less expensive at $15,654.

**Figure 2:**
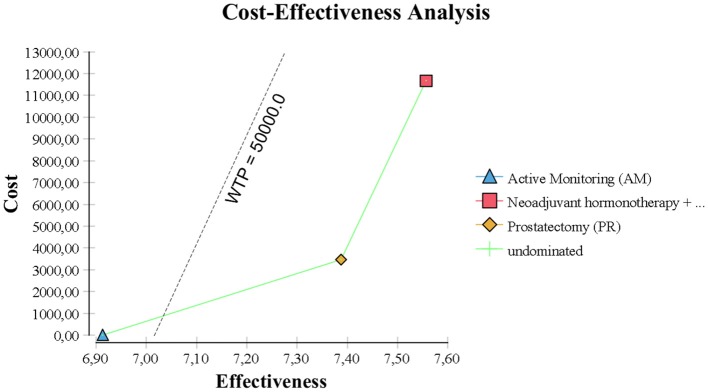
Incremental cost-effectiveness analysis.

However, AM was the best choice by ICER standards, because PR had an estimated ICER of $116,000 per life year gained (Figure 2). By US and UK standards, this is very expensive and probably unacceptable to most governments or insurance companies.

### Quality-Adjusted Life Years as an Effectiveness Measure

In the base case, RT provided the best quality-adjusted survival with an average of 7.61 QALYs in a 10 years model. PR was second-best at 7.44, and AM was least effective at 6.96. QALY differences were much greater than the life-year differences, to the extent that the ICER for PR vs. AM was only about $6500, making PR a good alternative to AM. PR was no longer the obvious choice over RT when QALYs were used, but the ICER for RT vs. PR was high at about $68,000 and within the threshold of about $50,000 to $100,000/QALY accepted by many US insurers. The results of the base-case analysis comparing AM, PR, and RT are presented in Tables 1, 2. Over 10 years, RT was 53.4% below the WTP threshold compared to AM, while PR was 85.4% below the threshold. AM was cost-effective at a WTP threshold of $1,000, PR at $1,500, and RT at $70,000.

**Table 1:** The results of the base case analysis.

	**Costs in $ (discounted 3%/year)**			**Life years (not discounted)**			**QALYs (not discounted)**		
	**Active monitoring**	**Prostatectomy**	**Radiotherapy**	**Active monitoring**	**Prostatectomy**	**Radiotherapy**	**Active monitoring**	**Prostatectomy**	**Radiotherapy**
Mean	15,654	18,791	30,378	9.54	9.57	9.57	6.96	7.44	7.61
Std Deviation	21,466	12,756	13,990	1.64	1.61	1.62	1.20	1.25	1.29

**Table 2:** Life years and QALYs as cost-effectiveness measures.

	**Active monitoring**	**Prostatectomy**	**Radiotherapy**
Cost effectiveness ($/LY)	Base	116,488	626,012
Cost effectiveness ($/QALY)	Base	6,548	68,339

### Sensitivity Analysis

**Figure 3:**
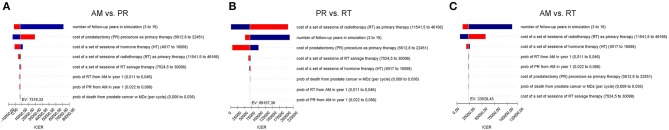
**(A)** Tornado diagram summarizing the results of one-way sensitivity analysis to identify model variables associated with the AM and PR in the treatment of localized prostate cancer. The influential factors are listed descending with the variation in value. **(B)** As **(A)** but for PR and RT. **(C)** As **(A)** but for AM and RT.

In order to test model responsiveness and result robustness, one-way sensitivity analysis was conducted. The variables in the sensitivity analysis varied from −50 to −200% of the base case values. The results are shown in Figure 3.

**Figure 4:**
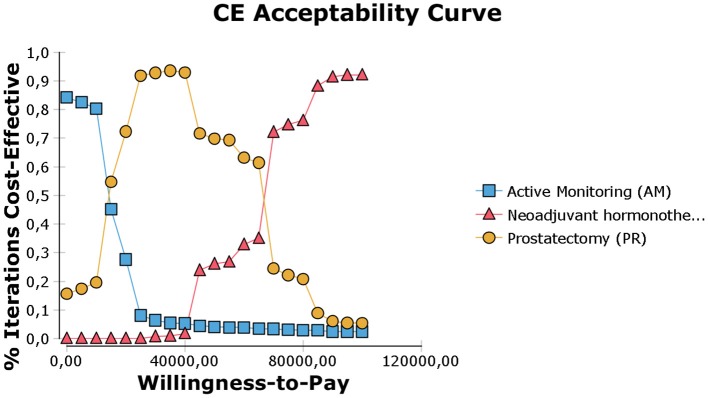
Cost-effectiveness acceptability curve (calculated with discounted incremental cost-effectiveness ratio (ICER) expressed as $/QALY. The WTP threshold corresponds to a given threshold ICER expressed as $/QALY.

The model was most sensitive to the number of years of follow-up, cost of procedure, and probability of metastatic disease, followed by cost of follow-up after PR and probabilities of death from other causes and salvage treatment. Only number of follow-up years and procedural costs decreased the RT vs. PR ICER below the WTP threshold of $50,000/QALY. Variation of the other values had little effect and resulted in ICERs that differed from the base case by <$10,000 per QALY. For follow-up years, the ICER was maximized in the first 3 years and then decreased up to the end of a trial observation period (Table 3). Of note, changes to RT cost had the greatest impact on the results of all the treatment-related costs. The probabilistic sensitivity analysis was considered using a cost-effectiveness acceptability curve and acceptability at WTP thresholds (Figure 4). At a threshold of $50,000/QALY, the probability of RT being cost-effective was 26% (Table 2). The cost-effectiveness acceptability curve also showed the probability of PR being cost-effective at a threshold limit of $70,000, and, at a threshold limit of $100,000/QALY, the probability of RT being cost-effective was 92.1%.

**Table 3:** One-way sensitivity analysis of years of follow up (costs in $).

**No. of years**	**Strategy**	**Cost**	**Incremental cost**	**Effectiveness**	**Incremental effectiveness**	**ICER**	**NMB**	**C/E**
2.0	AM^*^	3,848	0	1.45	0	0	−9,415	2,659
2.0	PR^**^	13,979	10,131	1.54	0.09	10,6332	−35,539	9,063
2.0	RT^***^	25,498	11,519	1.58	0.04	3,22,400	−65,736	16,158
4.0	AM	5,653	0	2.85	0	0	−21,786	1,981
4.0	PR	14,860	9,207	3.04	0.19	48,992	−60,060	4,885
4.0	RT	26,249	11,389	3.11	0.07	1,61,619	−1,07,945	8,433
6.0	AM	8,426	0	4.22	0	0.0	−44023	1995
6.0	PR	16,032	7,606	4.50	0.28	27,345	−88,222	3,561
6.0	RT	27,310	11,277	4.60	0.10	1,08,114	−1,53,127	5,928
8.0	AM	10,923	0.0	5.57	0.0	0	−71,717	1,962
8.0	PR	16,431	5,509	5.95	0.38	14,420	−1,14,165	2,763
8.0	RT	27,841	11,410	6.09	0.14	82,806	−1,97,276	4,575
10.0	AM	13,297	0.0	6.88	0.0	0.0	−1,04,758	1,933
10.0	PR	16,742	3,445	7.36	0.48	7,117	−1,39,996	2,274
10.0	RT	2,83,560	11,618	7.53	0.17	68,119	−2,41,981	3,765

* *Active monitoring*,

** *Prostatectomy*,

*** *Neoadjuvant hormonal therapy + radiotherapy*.

## Discussion

When different treatment methods have similar survival outcomes, health economics may support clinical and administrative decision-making on the most appropriate management. Here we assessed the cost-effectiveness of RT and PR in relation to AM using QALYs as the effectiveness measure. Over 10 years, with relaxed assumptions for AM, the ICER of PR and RT met the societal WTP threshold of $50,000 per QALY.

Prostatectomy and radiotherapy provide similar treatment efficacy at a higher cost during the early phases of treatment. However, these costs were balanced by better QoL than AM over the 10 years perspective. Whilst radical treatments resulted in reduced rates of metastases and disease progression, this was not shown to translate into a late survival benefit at 10 years, notwithstanding that further follow-up might reveal differences in survival benefit.

There are few cost-effectiveness analyses of different treatment modalities for prostate cancer. Earlier economic analyses were from the US (19–21) or Canadian (22) healthcare perspectives, the US cost-based analyses not including treatment of recurrences or side-effects and the other analyses excluding the costs of adverse effects.

Lao et al. (23) recently highlighted the impact of conversion from AM to PR. Approximately 20% of patients over first 2 years and 50% of patients over 10 years will progress to more aggressive cancer and subsequently undergo curative intervention, most commonly with surgery or radiotherapy (3, 4, 10, 23, 24). With this in mind, AM was less likely to be cost-effective compared to radical prostatectomy for younger men diagnosed with low-risk localized prostate cancer, with an estimated 5% conversion rate from AM to PR. With an annual conversion rate of 1.6%, life-time costs of AM were lower than the costs of radical prostatectomy for men aged 55–70 (23).

We assumed that the probability of having treatment annually in the AM arm was 13% in the first year and 5% in consecutive years, assuming that the conversion rates reported in the ProtecT trial made our analysis more realistic. Further, in Lao et al.'s study (23), the AM arm only considered radical prostatectomy as a treatment option, which may decrease the real cost of AM. Taking radiation and surgery as definitive treatments into account could be considered a strength of the current analysis.

Similar studies have been affected not only by the possibility of having radical prostatectomy when managed with AM but also uncertainties around good QoL data for men under AM. We used Markov decision analysis modeling of ProtecT trial data to assess QALYs from the 10 years perspective, as it was the first prospective trial with QoL life data on all three management strategies. Earlier studies (5, 25) based on the PIVOT (12) and SPCG (26) trials reported different results. In an analysis by Hayes et al. (5), AM was associated with improved QALYs compared with initial treatment. Further, in a German study (25), AM was superior to initial treatment with higher QALYs. In this case, costs were included from the German health service perspective and substantially differed from US costs. Moreover, probabilities were taken from trials comparing PR with watchful waiting, the latter representing a different strategy to AM in ProtecT, in that watchful waiting tends to be reserved for older men with significant medical comorbidities who are likely to suffer decreased QoL with aggressive treatment. However, in contrast to watchful waiting, an AM protocol advocates a potential intention to treat and therefore imposes often rigorous follow-up with frequent PSA measurements, office visits, and prostate biopsies.

Our model was sensitive to the probability of developing metastases under AM, similar to reported previously (25). At a time horizon of 2.5 years, conservative management was preferable to radical prostatectomy in terms of costs in a claims data analysis (27), consistent with our data showing that cost-effectiveness is very sensitive to follow-up time and was not a cost-effective approach over short periods of observation. Thus, AM should be a reasonable option for patients with shorter life expectancy.

Our AM strategy attempted to reproduce the ProtecT protocol but was modified slightly to reach current NCCN recommendations. According to NCCN, PSA should be assessed every 6 months from the beginning of monitoring, while in ProtecT it was every 3 months in year one and every 6–12 months thereafter (8). Regardless, sensitivity analysis showed little impact of PSA test costs on ICERs. In a recent cost-effectiveness analysis of active surveillance strategies for men with low-risk prostate cancer (28), a similar strategy was compared with MRI incorporation into surveillance protocols, which was found to be cost effective; however, this was not used in ProtecT so was not considered here.

Our results are in line with Cooperberg et al. (6), which showed substantial payer and patient costs when radiotherapy was used. In a recent analyses utilizing time-driven activity-based costing (29, 30) brachytherapy and stereotactic body radiotherapy were notably cheaper radiation modality and alternative to 3D conformal radiotherapy used in ProtecT. However, attending physician may work 1.6–3.4x more time per relative value unit when delivering brachytherapy compared to intensity-modulated radiotherapy (IMRT) (31). This resulted that contemporary practice usually involves the more costly but less intensive and non-invasive IMRT (31). Recent cost-effectiveness studies have shown that SBRT is an attractive alternative to IMRT (32, 33), with SBRT cost savings attributable to shorter procedure times and fewer visits required for treatment. This may be especially attractive in terms of cost-effectiveness, as ICERs could decrease below a critical WTP threshold. If used routinely, SBRT should increase QALYs or decrease costs. Our cost-effectiveness acceptability curve suggested that SBRT (cost $11,665) could be superior to the alternatives, but only if it results in a similar QoL. Precise evaluation of SBRT QoL compared to RT may play a crucial role in future early prostate cancer management.

Based on SEER data, the incidence of prostate cancer in the US is expected to reach 160,000 new cases per year (1). Due to this high incidence, the cost savings for AM would amount to hundreds of billions of dollars per year, so the willingness to pay for a QALY in this large population needs careful assessment.

This analysis was based on effectiveness, risk of complications and adverse events, progression, cancer, and non-cancer related deaths, and QoL data from the first prospective, randomized study of three management alternatives and adhering to cost-effectiveness analysis standards. However, because ProtecT excluded patients >69 years of age or with PSAs >20 ng/ml or PSAs 10–20 ng/ml without a bone scan performed, our results should be interpreted with caution in such groups.

There are several important limitations to this study. According to standard practice guidelines, androgen deprivation therapy or antiandrogen therapy should not be used routinely in low and favorable intermediate risk localized prostate cancer (8). Our cost analysis is based on a model that used published data not source data, so progression rates may reflect deficiencies in the literature used. In this context, men who progressed on AM received either PR or RT based on our assumptions and understanding of the published data, and we deliberately excluded brachytherapy or cryotherapy due to the lower popularity of these therapies and to simplify this model. The procedure costs were from Medicare 2008 and may differ from today's prices; additionally, some model inputs relied on expert opinion and may differ between institutions. However, sensitivity analysis was performed to assure the robustness of the findings. The probabilities were fit to males aged 50–69 with at least 10 years life expectancy and may not be easily generalizable to other populations (34). Further, our study used summary rather than individual patient data from a randomized trial, and summary data limits the unexpected rate of differences. Also, to avoid influence, trials results are never free from factors affecting generalizability, and trial-based cost analyses inherit these limitations (35). However, the strength of modeling through decision is to address the problem of generalizability of clinical trial results to real-world settings and alleviate problems associated with the inclusion of protocol-driven costs (7). In contrast to cost analyses based on raw data from clinical trials, we focused only on the costs occurring for a clinical reason (7).

The strength of this paper was transferring all outcomes and costs to the US payer perspective independent of the location in which the original trial was undertaken. To our knowledge, this is the first cost-effectiveness evaluation of ProtecT. The model can be considered an abstraction of a trial by synthesizing information from multiple sources to provide decision makers with the best available evidence to reach a decision (36).

In conclusion, prostatectomy or radiotherapy prevented decreased QoL and did so at a cost that was below common willingness-to-pay thresholds. These results were robust to extensive sensitivity analyses.

## Data Availability Statement

The datasets generated for this study are available on request to the corresponding author.

## Author Contributions

AH and MH coordinated and performed data analyses, reported study results, and drafted the manuscript. MM performed analyses. MH was the clinician responsible for the interpretation of clinical data. All authors read and approved the final manuscript.

### Conflict of Interest

MM was employed by company Technomics Research LLC. The remaining authors declare that the research was conducted in the absence of any commercial or financial relationships that could be construed as a potential conflict of interest.
