# Supplementary material for: A Cost-Effectiveness and Quality of Life Analysis of Different Approaches to the Management and Treatment of Localized Prostate Cancer
Source: Front Oncol. 2020 Feb 11;10:103. doi: 10.3389/fonc.2020.00103 (PMC7026676; doi:10.3389/fonc.2020.00103)
Supplement: Supplementary file 1 [file Table_1.DOCX]

Supplementary Material

**Supplementary Table 1.** Model parameters and assumptions.

| **Variable** | **Definition** | **Value used in model** |
| --- | --- | --- |
| c_biopsy | cost per stage of AM (continues until treatment) | 249.50 |
| c_DxCP | cost of Dx of CP | 1254.00 |
| c_HT | cost per stage of HT (continues for life) | 8034.00 |
| c_HT_FU | cost per stage of HT FU (continues for life) | 1254.00 |
| c_PR | cost per procedure of PR (primary or salvage) | 11225.67 |
| c_PR_FU | cost of PR FU | 1640.34 |
| c_PSAtest | cost per PSA test | 124.00 |
| c_RT | cost per set of sessions of RT as primary therapy | 23083.00 |
| c_RT_FU | cost of RT FU | 1522.10 |
| f_PSAtest_yr1to4 | frequency of PSA test in 1/2 years (stages 1 to 8) | 1.00 |
| f_PSAtest_gtyr4 | frequency of PSA test after stage 8 (year 4) | 0.50 |
| n_years | number of years in simulation | 10.00 |
| p_AM_MDz_gtyr1 | in AM, prob of moving to MDz after year 1 (per stage) | 0.0051 |
| p_AM_MDz_yr1 | in AM, prob of moving to MDz in year 1 (per stage) | 0.0000 |
| p_AM_PR_gtyr1 | in AM, prob of moving to PR after year 1 (per stage) | 0.0028 |
| p_AM_PR_yr1 | in AM, prob of moving to PR in year 1 (per stage) | 0.0440 |
| p_AM_RT_gtyr1 | in AM, prob of moving to RT after year 1 (per stage) | 0.0021 |
| p_AM_RT_yr1 | in AM, prob of moving to RT in year 1 (per stage) | 0.0229 |
| p_Death_Other | prob of death from other causes (per stage) | 0.0046 |
| p_Death_PrCA | prob of death from PrCA \| MDz (per stage) | 0.0179 |
| p_PR_MDz_gtyr1 | after PR, prob of moving to MDz after year 1 (per stage) | 0.0013 |
| p_PR_MDz_yr1 | after PR, prob of moving to MDz in year 1 (per stage) | 0.0010 |
| p_PR_RT_gtyr1 | after PR, prob of moving to RT after year 1 (per stage) | 0.0037 |
| p_PR_RT_yr1 | after PR, prob of moving to RT in year 1 (per stage) | 0.0028 |
| p_RT_MDz_gtyr1 | after RT, prob of moving to MDz after year 1 (per stage) | 0.0017 |
| p_RT_MDz_yr1 | after RT, prob of moving to MDz in year 1 (per stage) | 0.0012 |
| p_RT_PR_gtyr1 | after RT, prob of moving to PR after year 1 (per stage) | 0.0035 |
| p_RT_PR_yr1 | after RT, prob of moving to PR in year 1 (per stage) | 0.0025 |
| e_life_years | effectiveness of treatment in life years (per 1/2 year) | 0.50 |
| c_RT_salvage | cost per set of sessions of RT as salvage therapy | 15049.00 |
| c_mgmt_GIlong | cost (semi annual) of management of long-term GI problems (after year 2) | 722.00 |
| c_mgmt_GIshort | cost (semi annual) of management of short-term GI problems (through year 2) | 577.00 |
| c_mgmt_INC | cost (semi annual) of management of incontinence (a side effect) | 461.00 |
| c_mgmt_SexDfn | cost (semi annual) of management of sexual dysfunction (a side effect) | 245.50 |
| c_mgmt_Usympt | cost (semi annual) of management of urinary symptoms (a side effect) | 97.50 |
| p_AM_Inc | in AM, prob of getting incontinence (per stage) | 0.0054 |
| p_AM_Sdysfn | in AM, prob of getting sexual dysfucntion (per stage) | 0.0256 |
| p_AM_Usymp | in AM, prob of getting urinary symptoms (per stage) | 0.0248 |
| p_PR_INC | after PR, prob of getting incontinence (per stage) | 0.2199 |
| p_PR_Sdysfn | after PR, prob of getting sexual dysfunction (per stage) | 0.2698 |
| p_PR_Usymp | after PR, prob of getting urinary symptoms (per stage) | 0.0754 |
| p_RT_GIlong | after RT, prob of getting GI problems (long-term) (per stage) | 0.0356 |
| p_RT_GIshort | after RT, prob of getting GI problems (short-term) (per stage) | 0.0247 |
| p_RT_INC | after RT, prob of getting incontinence (per stage) | 0.0027 |
| p_RT_Sdysfn | after RT, prob of getting sexual dysfunction (per stage) | 0.2049 |
| p_RT_Usymp | after RT, prob of getting urinary symptoms (per stage) | 0.0457 |
| qol_AM | QALYs per life year with AM (per stage) | 0.3645 |
| qol_PR | QALYs per life year with PR (per stage) | 0.3885 |
| qol_RT | QALYs per life year with RT (per stage) | 0.3975 |
